# A cross-sectional examination of the early-onset hypertensive disorders of pregnancy and industrial emissions of toxic metals using Kentucky birth records, 2008–2017

**DOI:** 10.1371/journal.pone.0274250

**Published:** 2022-09-20

**Authors:** Courtney J. Walker, W. Jay Christian, Anna Kucharska-Newton, Steven R. Browning

**Affiliations:** Department of Epidemiology, College of Public Health, University of Kentucky, Lexington, Kentucky, United States of America; King Faisal Specialist Hospital and Research Center, SAUDI ARABIA

## Abstract

This cross-sectional study assessed geospatial patterns of early-onset hypertensive disorders of pregnancy (eHDP) in primiparous mothers and exposure to industrial emissions using geocoded residential information from Kentucky live (N = 210,804) and still (N = 1,247) birth records (2008–2017) and census block group estimates of aerosol concentrations of arsenic (As), cadmium (Cd), chromium (Cr), lead (Pb), mercury (Hg), selenium (Se), and zinc (Zi) from the Risk Screening Environmental Indicators (RSEI) model. A latent class analysis allowed for the identification of four district exposure classes—As, Cd, and Pb (12.6%); Se and Zi (21.4%); Pb and Cr (8%); and low or no exposures (57.9%). Women classified as having a high probability of exposure to both Pb and Cr had a statistically significantly greater prevalence of eHDP after adjusting for demographic factors (aPR = 1.22, 95% CI: 1.04, 1.44) relative to those with low or no exposure. Our findings contribute to the emerging literature on the association of metal exposures with pregnancy outcomes.

## Introduction

Exposure to environmental toxicants has been shown to increase the risk of respiratory and cardiovascular disease, breast cancers, and poor pregnancy outcomes such as hypertensive disorders of pregnancy (HDP) [1–3]. Hypertensive disorders of pregnancy impact 8–10% of pregnancies in the U.S. each year and are among the leading causes of morbidity and mortality in mothers and infants. Subsets of HDP include gestational hypertension (GH), pre-eclampsia (PE), HELLP (Hemolysis, ELevated Liver enzymes, Low Platelet count), and eclampsia [4, 5]. Short-term adverse events of HDP can include preterm birth, maternal stroke, or maternal renal failure. Even after the resolution of the pregnancy, women who experience more severe HDP, such as PE and HELLP, are at an increased risk of hypertension, stroke, metabolic disease, and HDP in subsequent pregnancies [6, 7]. Pharmacologic interventions and treatments are extremely limited [8]. Factors that increase the risk of HDP include primiparity, maternal age, obesity, race, and the use of infertility treatment [9–15]. Preliminary research suggests maternal exposure to trace elements may also be associated with an increased risk of HDP.

The assessment of trace element exposure on maternal health has largely been inconclusive or needs further study, particularly in assessing the co-occurrence of trace elements. However, studies assessing a limited number of trace elements have found that in mice, cadmium exposure is associated with increased blood pressure, proteinuria, and intrauterine growth restriction (IUGR)–the hallmarks of PE [16]. In a case-control study of human subjects, Laine and colleagues found that PE cases had higher cadmium levels and lower selenium levels than controls [17]. In one of the few studies assessing the co-occurrence of other trace elements (selenium), researchers reported that the odds of PE were 50% higher among those who had higher cadmium exposure; assessments of interactions of cadmium and below medians exposure to selenium also increased the odds of PE. Lead has also been found to be associated with chronic hypertension and PE. In a recent literature review, Poropat and colleagues concluded that lead exposure is one of the most important risk factors for PE yet identified [18]. Although more work remains, evidence suggests that lead may induce vasoconstriction and placental ischemia—thus inducing hypertension and proteinuria—notable events that occur during the second stage of PE [19]. As and Hg studies have been inconclusive; however, both have been linked to cardiovascular disease [20] and pregnancy loss and complications [21]. Other PE case-control studies have found PE cases had substantially higher levels of lead and As [22] as well as Hg [23] and Cr [18].

Assessing the synergistic impact of the co-occurrence of chemical exposure has had limited exploration in pregnant humans. Further, these studies have occurred in locations with a wide variety of regulations in environmental regulations and infrastructure (Mexico, US, China, the Democratic Republic of the Congo, etc.). Although these studies are diverse and important, they create challenges in generalizing to other populations, as infrastructure and regulationscan impact the overall risk of exposure and the amount that a pregnant person would have been exposed to [24]. One potential option to address this limitation is to use the Environmental Protection Agency’s (EPA) Risk Screening Environmental Indicators (RSEI) model [25]. The (RSEI) approach uses emissions data reported to the Toxic Release Inventory (TRI) program to characterize yearly ambient aerosol concentrations of individual chemicals of concern across the U.S., adjusted for physicochemical properties and site characteristics (such as stack height, when available) [25]. These data are attractive, particularly in pilot studies, as they are easily accessible, include over 700 chemicals tracked by the EPA, are weighted for their overall toxic effects on human health, and can be linked to administrative boundaries, such as zip code tabulation areas (ZCTA), census tracts, or block groups [25].

To assess the utility of RSEI data, we selected birth records from Kentucky (2008–2017), a state with a high prevalence of adverse eHDP risk factors (obesity, pre-existing diabetes) [26] and a high prevalence of smoking, which has generally been found to be protective against developing eHDP [27]. Kentuckyalso hosts large industrial facilities in both urban and rural areas, making it well suited to explore the relationship between environmental metal exposures and eHDP [28]. In this study, we had four aims: 1) examine the distribution of emissions of chemicals of concern across the state, 2) identify patterns of exposure to industrial metal emissions and describe the sociodemographic characteristics of mothers in these areas, 3) evaluate the impact of environmental exposures to industrial metal emissions, adjusting for sociodemographic factors on risk eHDP, and 4) identify areas in the state that have a high prevalence of individuals with eHDP. We hypothesized that women living in areas with an overlap in exposures associated with HDP, such as As, Cr, Cd, and Pb during pregnancy would have a higher probability of eHDP than those living in areas with singular exposures. We also expected that women who lived in areas with elevated Se or Zn concentration would be less likely to have an eHDP diagnosis, as Se and Zn have been shown to be protective against the effects of Cd toxicity [17].

## Methods

The Kentucky Cabinet for Health and Family Services and the University of Kentucky Medical Institutional Review board reviewed and approved this cross-sectional study protocol (Protocol 44968, Approved 10/26/2018). As this study accessed data routinely collected in birth certificates, the IRB waived the requirement for informed consent and did not require participants to provide written consent. While they did not contain names, medical record numbers, or social security numbers, these data were not fully anonymous as they included full addresses for all births. Birth Records data are not publicly available but may be requested from the Commonwealth of Kentucky’s Community for Health and Family Services. branch [29] Strengthening the Reporting of Observational Studies in Epidemiology (STROBE) guidelines were used as a reporting template [30].

### Study population

The Kentucky Department of Vital Statistics provided 557,751 individual records for all live (n = 553,476) and stillbirths (n = 3,268) to self-identified Kentucky residents from January 1, 2008, through December 31, 2017. All covariates assessed for model inclusion were present in both forms [31]. This time was selected to provide a 10-year window of trends. The end of the study period corresponded to the last full year that data was available at the time of the request (November 2018).

Non-singleton records (n = 5,206) with non-primiparous (n = 327,459) mothers younger than 11 years or greater than 50 years were excluded (n = 215). Further, records indicating the mother had chronic hypertension, as it is mutually exclusive with HDP on birth records (n = 10,752), [32] or delivered before 20 weeks gestation or after 45 weeks (n = 565), were excluded. In addition, records were excluded if the sex of the child was not known (n = 20), the record did not geocode (n = 3), or geocoded outside of the state (n = 473), leaving 212,051 (1,247 stillbirths and 210,804 live births) for analysis.

Early-onset hypertension (eHDP) was defined as giving birth at or before 34 weeks and being positive for GH, which includes diagnoses of GH, PE, or HELLP on the birth record [32]. Individual-level data were assessed for patterns in missingness using PROC MI in SAS [33]. There was no indication of bias with missing information.

Covariate definitions The publicly available (2013) Rural-Urban Continuum Codes (RUCC) were obtained from the United States Department of Agriculture to characterize the intensity of urban development in each county [34]. The Area Deprivation Index (ADI), also publicly available, was used to characterize economic distress by census tract [35]. The ADI dataset incorporates American Community Survey (ACS) data on income, housing, educational, and employment data into principal component analysis to derive a score that is then standardized across each state. Higher scores correspond to higher levels of economic distress [36]. For this study, the 2019 Kentucky-specific census block group ADI data were linked to the birth records by the geocoded census block group (CBG). The ADI scores were dichotomized into upper quintiles (ADI of 9–10) and lower (ADI 1–8) of neighborhood poverty [35]. Cartographic boundary files for county [37] and census block [38] were obtained from the United States Census Bureau. Appalachian status, defined by the Appalachian Regional Commission (ARC), was based on the geocoded maternal county of residence [39].

#### Outcome and exposure ascertainment

To assess environmental exposure, disaggregated CBG air emissions data were requested and obtained from the Environmental Protection Agency’s (EPA) Risk Screening Environmental Indicators (RSEI) program for the years 2007 through 2017 [40]. Briefly, RSEI uses the yearly chemical-specific stack and fugitive aerosolized emissions reported to the Toxic Release Inventory (TRI) by federal and mandated facilities. Facilities are required to report if: 1) they have ten or more full-time employees, 2) the industry is in a required sector (such as mining) or is a federal facility, and 3) manufactures, processes, or uses TRI-listed chemicals, and the production, use, or transfer of a chemical amount exceeds the threshold set for a chemical in a given category [41]. Although some RSEI data is publicly available, we requested CBG from the EPA [40].

The PROC RANK procedure in SAS was used to identify and classify the highest quintiles for each exposure [42]. As few CBG had an estimated toxicity concentration of Cd, Cd exposure was dichotomized into yes or no, as Cd is a noted risk factor for HDP [17]. The yearly RSEI data were linked to birth records by CBG using the residential address and year the majority (>50%) of the pregnancy period took place, including a 12-week preconception period to account for pre-pregnancy exposures [43, 44]. Therefore, although birth records were from 2008–2017, we used exposure data from 2007–2017.

#### Data cleaning and dataset preparation

Individual-level covariates were obtained from the birth records. Maternal age was calculated by subtracting the mother and infant’s dates of birth and rounding down to the nearest year, then categorized into five groups (≤20, 21–23, 24–28, 29–34, ≥35 years). Maternal race was collapsed into three categories (Black, Other, White). Maternal Body Mass Index (BMI) was calculated using self-reported height and pre-pregnancy weight and categorized into: underweight (<18.0 kg/m^2^), normal (18–24.9 kg/m^2^), overweight (25–30 kg/m^2^), and obese (≥30 kg/m^2^) [45, 46]. Current literature suggests that women who quit smoking during their first trimester have an equivalent risk of HDP as women who do not smoke. Therefore, those who reported no smoking throughout their pregnancy or reported no cigarette use after the second trimester were considered non-smokers. Otherwise, women were classified as smokers [47]. Other covariates captured on the birth record included maternal ethnicity (Hispanic/non-Hispanic), education (less than high school, high school, some college, and college degree), marital status (yes/no, or not stated), and pre-existing diabetes (yes/no, or not stated).

Each record was geocoded using an ESRI address coder (ESRI, Redlands, CA). This process provides geographic coordinates, the precision of coordinates, and local administrative boundaries (county and census tract information) for each address. Precisely geocoded addresses were those that were identifiable at the rooftop or by street address range. Addresses geocoded to the centroid of a street, city, or ZIP code were considered imprecise.

#### Statistical analysis

To assess geospatial patterns of disease, SaTScan software was used to conduct a retrospective spatiotemporal scan statistic (Bernoulli model) with an elliptical scan window to detect clusters of high eHDP rates in Kentucky over the study period [48]. SaTScan^™^ is a trademark of Martin Kulldorff. The SaTScan (TM) software was developed under the joint auspices of (i) Martin Kulldorff, (ii) the National Cancer Institute, and (iii) Farzad Mostashari of the ew York City Department of Health and Mental Hygiene [48].

This method identifies candidate clusters using overlapping cylinders of increasing heights and diameters representing time and spatial dimensions, respectively until a user-defined maximum population (10%) and temporal inclusion (5 years) restriction is reached. The maximum population is defined by the input file’s number of cases and non-cases. Using the likelihood ratio test, SaTScan compares the number of observed and expected cases within a candidate cluster to the area outside the cluster, adjusting for the underlying population. The Monte Carlo method (999 simulations) was used to estimate the p-value [48].

We assessed purely spatial candidate clusters, those that spanned the entire study period and clusters that encompassed 50% or less of the study period [48]. To identify high-rate clusters of eHDP, the maximum cluster size was restricted to 10% of the population after confirming that larger population centers such as Lexington, Kentucky, and Louisville, Kentucky had no clusters, as population size restrictions would effectively exclude them from reported results.

*Latent Class Analysis (LCA)*. The PROC LCA macro, developed by Lanza and colleagues, was used to conduct the latent class analysis [49]. In this analysis, the goal was to identify homogenous subgroups characterized by a combination of environmental emissions exposures using the dichotomized CBG estimates of exposure. Zinc (Zn) and Selenium (Se) were also included, given recent findings that suggest these trace elements can moderate or reverse the impact of cadmium on the risk of HDP [17]. To determine the most appropriate class structure, following the guidance from Lanza, ten sets of models with random starting values consisting of two to five classes were run. When 80% of the models converged to the same solution, we felt confident that we had identified the right model.[49] Model fit was assessed using the Akaike information criterion (AIC), Bayesian information criterion (BIC), entropy, visual distinctiveness of each class, and class size [49, 50]. Specifically, models with the highest entropy, low AIC and BIC, and class sizes greater than five records were prioritized.

*Statistical modeling*. To assess the distribution of demographic characteristics, we summarized the overall sample with frequencies and percentages and provided row percentages for each demographic subgroup by exposure class. We used counts and column percentages to summarize the prevalence of eHDP by sociodemographic factors. A bivariate logistic regression was fit to examine the relationship between eHDP and covariates (sociodemographic factors and environmental exposure class). Variables were selected for inclusion into the final model, a multivariable logistic regression, if they were noted risk factors for eHDP (maternal age, race, obesity, pre-existing diabetes, smoking), (9) were exposures of concern (As, Cd, Cr, Hg, and Pb), or were associated with eHDP in the bivariate logistic regression (mother’s ethnicity, education, ADI, Appalachian region, RUCC status, and stillbirth). Geocoding precision was included to adjust for geocoding misclassification. The final model consisted of latent class groups maternal demographic characteristics [age (years), race, and ethnicity]; maternal health characteristics [BMI, pre-existing diabetes, and smoking through pregnancy] and community characteristics [ADI and the Appalachian region]. To assess potential biases resulting from using the latent class assignment as a categorical variable, we conducted an additional multivariable logistic regression with a subset of participants with a posterior probability (PPr) of less than 80%. The proportion of records in each class with a PPr of less than 80% is summarized in Appendix A.

SAS v 9.4 (SAS Institute, Cary, NC) was used for all non-spatial statistical analyses. P-values less than 0.05 were considered statistically significant.

## Results

### Spatial analysis

**Fig 1:**
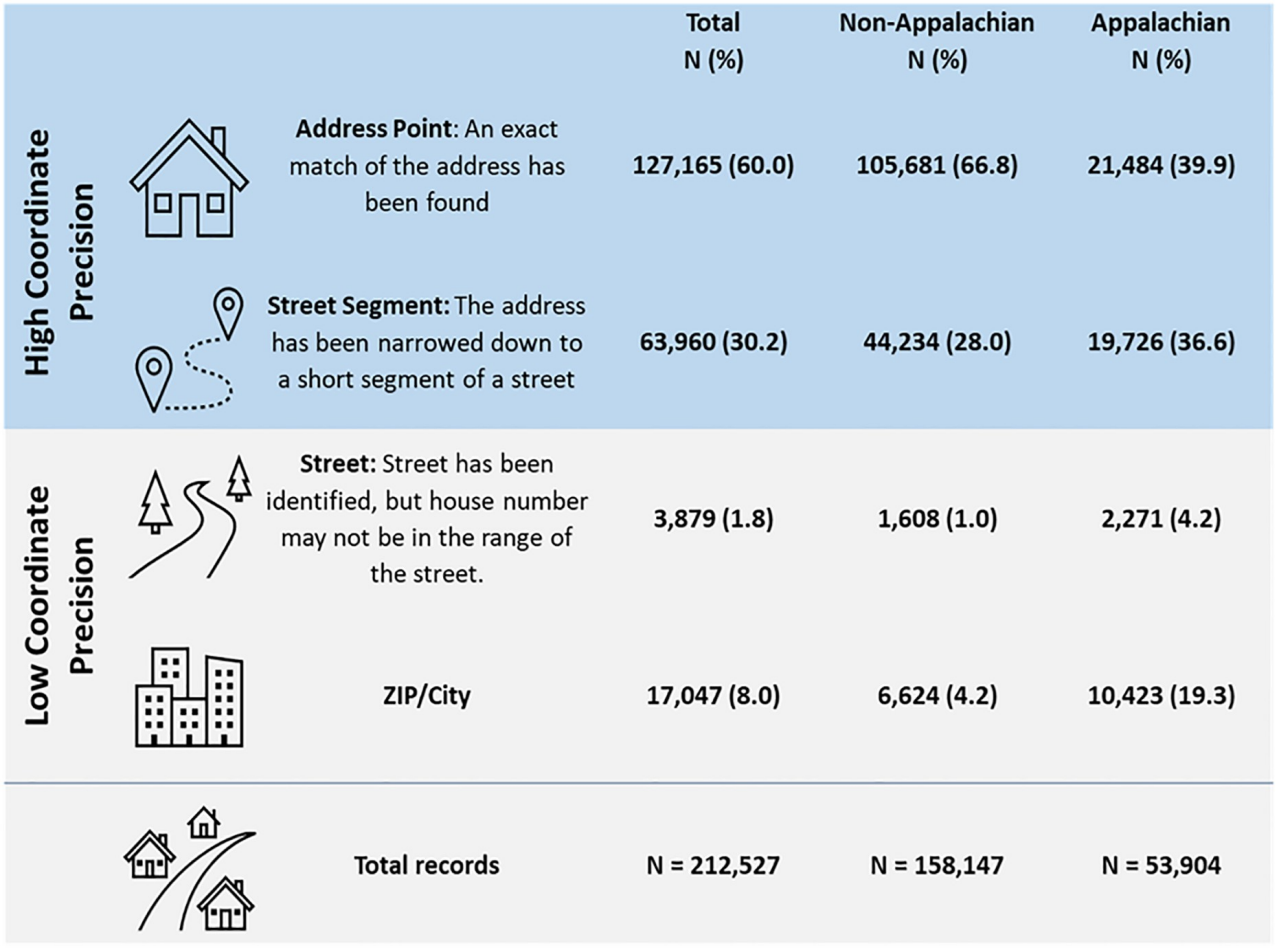
Description and summary of geocoding precision for Kentucky addresses, 2008–2017.

Fig 1 presents the description and geocoding precision for Kentucky addresses used in this study. Overall, the majority of address records were geocoded with high precision (80%). In the non-Appalachian region, 94% of addresses geocoded to either a street segment (28%) or address point (67%). Almost 77% of addresses had high coordinate precision in the Appalachian region.

**Fig 2:**
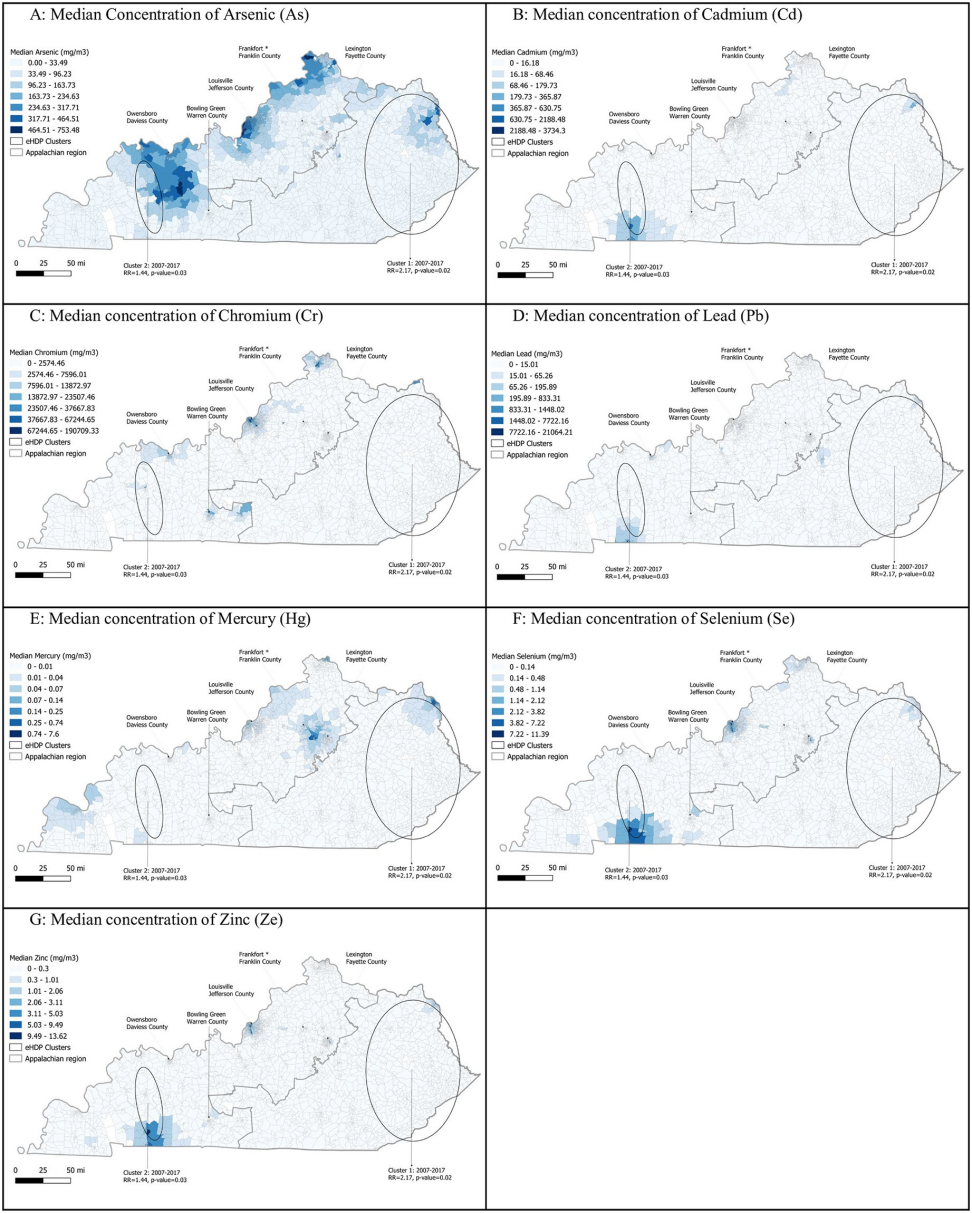
Median toxicity concentrations of emissions for each census block group, Kentucky 2007–2017.

Fig 2 consists of a series of maps that display two individual level clusters of eHDP and choropleth maps of the median emissions for each chemical over the study period. Women living within the largest cluster, located within the Appalachian region, had a 117% greater risk of eHDP than women outside the cluster (RR = 2.17, p-value, 0.02). The second cluster, in Western Kentucky, was smaller, but women within this cluster had a 44% greater risk of eHDP compared to women outside of the cluster. (RR = 1.44, p-value = 0.03). Both clusters were significant for the entire duration of the study period (2007–2017). We also observed that, compared to other regions of the state, Louisville, Kentucky’s largest city, had high median concentration estimates of As, Cr, Se, and Zn. The state’s southwestern border (near Hopkinsville) and elevated median emissions of Zn, Se, and Cd. Most of the Appalachian region in eastern Kentucky had low emission medians over the study period.

### Latent Class Analysis (LCA)

Table 1 summarizes the model fit characteristics for LCAs with 2–4 classes. We chose the four-class model as the groups were distinctive, all classes had greater than five records, and the AIC and the BIC were the lowest of the four models, although the entropy was slightly lower than the 2-class model.

**Table 1:** Indicators of fit for latent class analysis with two through four classes of emission exposures.

Number of Classes	AIC	BIC	Entropy
2	85797.7	85950.9	0.95
3	34648.3	34884.1	0.86
4	7388.4	7706.6	0.89

**Fig 3:**
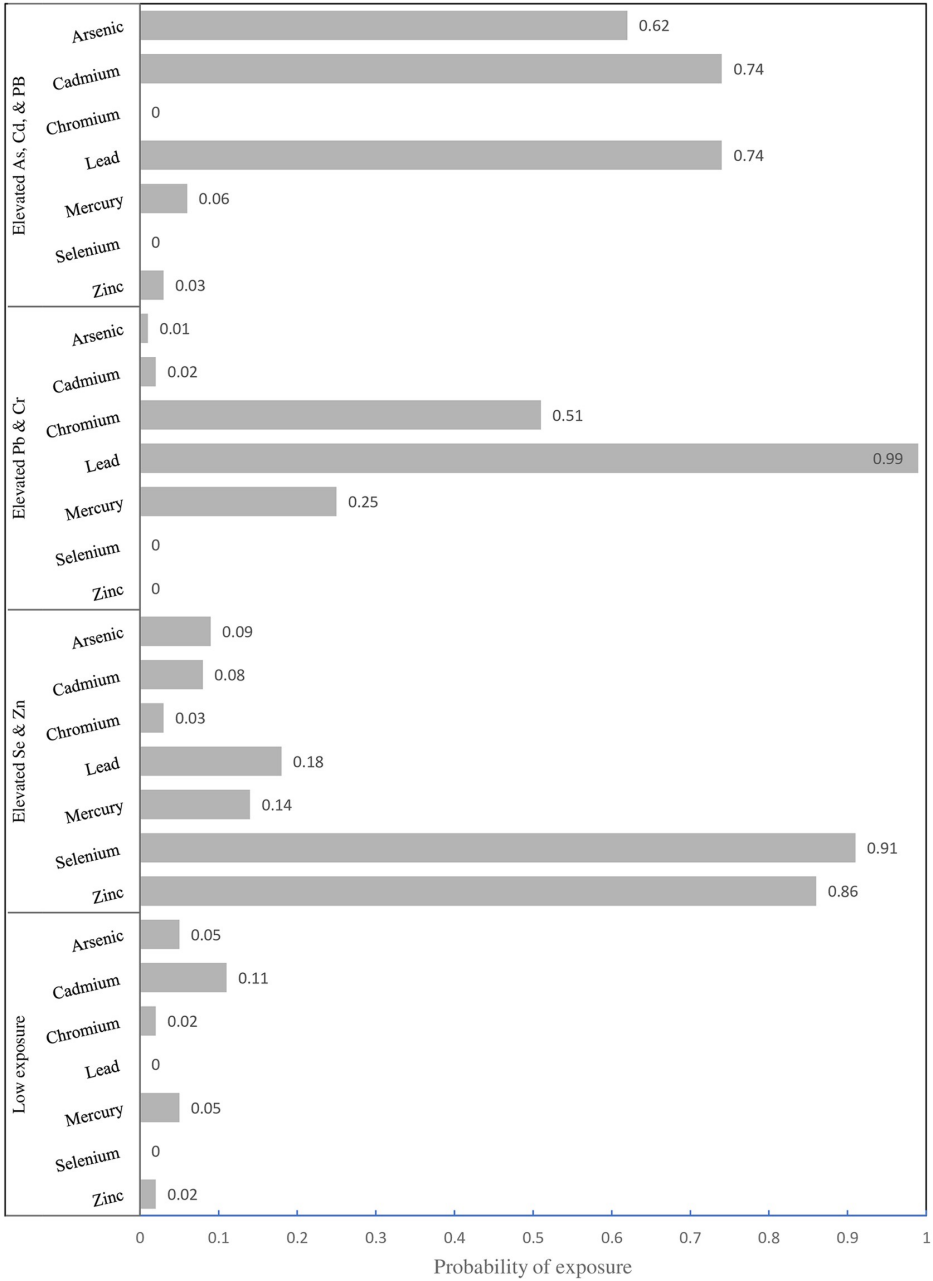
Class membership probabilities as a function of environmental chemical exposure.

LCA: Latent Class Analysis, AIC: Akaike’s Information Criteria,

BIC: Bayesian Information Criteria;

Fig 3 visualizes the item response probabilities for each class. The first class (N = 26,773) had a 74% probability of elevated Cd exposure, 62% probability of elevated As exposure, and a 74% probability of Pb exposure. Women in the second class (N = 45,391) had a high probability of living in an area with both Se (91%) & Zn (86%) exposure. Those in the third exposure class (n = 17,058) had a 99% probability of Pb exposure and a slightly elevated probability of Cr (51%). The final class, which constituted the majority of first-time mothers (n = 122,829), had almost no metal exposure.

### Statistical modeling

Table 2 describes the demographic characteristics of the study population overall and by latent class. First-time mothers were predominantly non-Hispanic (96%), White (85%), 13–20 years old (30%), with a BMI between 18.0–24.9 (kg/m^2^) (45%), and largely non-smoking (86%).

**Table 2:** Demographic characteristics summary by latent metal class and the total population of primiparous mothers 2008–2017.

	Total	Elevated As, Cr & Pb	Elevated Se & Zn	Elevated Pb & Cr	Low Exposure
	205836 (100%)	26773 (12.6%)	45391 (21.4%)	17058 (8.0%)	122829 (57.9%)
	N (%)	%	%	%	%
**Mother’s age (years)** †					
≥35	10054 (4.7)	13.9	22.1	7.4	56.7
29–34	37256 (17.6)	13.7	22.7	7.7	55.9
24–28	58053 (27.4)	12	22.8	8.2	57
21–23	43175 (20.4)	11.9	20.7	8.5	58.9
≤20	63513 (30.0)	12.8	19.8	7.9	59.5
**Mothers race** †					
Black	17512 (8.3)	31.4	14.8	10.1	43.7
Other	14222 (6.7)	18.8	21.0	8.2	51.9
White	180317 (85.0)	10.3	22.1	7.8	59.8
**Mother’s ethnicity** †					
Non-Hispanic	203521 (96.0)	12.5	21.3	8.0	58.2
Hispanic	8530 (4.0)	16.3	24.2	8.0	51.4
**Mother’s education** †					
College Degree	69526 (32.8)	12.6	23.5	7.9	56.0
Some College	49760 (23.5)	13.2	21.5	8.8	56.5
Less than High School	32824 (15.5)	14.8	18.5	7.6	59.1
Unknown	1923 (0.9)	14.5	21.6	6.0	57.9
High School	58018 (27.4)	10.8	20.5	7.9	60.8
**Mother’s BMI (kg/m^2^)** †					
Obese (≥30.0)	49608 (23.4)	11.6	20.9	7.4	60.1
Overweight (25.0–29.9)	50122 (23.6)	12.4	21.3	7.9	58.4
Underweight (<18.0)	11309 (5.3)	12.2	20.6	8.3	58.9
Unknown	6215 (2.9)	18.9	22.7	7.8	50.6
Normal (18.0–24.9)	94797 (44.7)	12.9	21.7	8.5	56.9
**Pre-existing diabetes** †					
Yes	1608 (0.8)	10.8	21.3	7.2	60.8
No or not stated	210443 (99.2)	12.6	21.4	8.1	57.9
**Smoking throughout pregnancy** †					
No	178231 (84.1)	13.0	21.7	8.1	57.2
Unknown	3783 (1.8)	12.7	22.4	7.5	57.5
Yes	30037 (14.2)	10.5	19.6	7.8	62.2
**Mother married** †					
Yes	107478 (50.7)	10.7	22.5	8.6	58.3
No or not stated	104573 (49.3)	14.6	20.3	7.5	57.5
**Stillbirth** †					
Yes	1247 (0.6)	14.4	18.6	7.3	59.7
No or not stated	210804 (99.4)	12.6	21.4	8.0	57.9
**Geocoding precision** †					
Address point/Street segment	189367 (89.3)	13.5	22.3	8.3	55.9
Imprecise**	22684 (10.7)	5.7	13.8	5.9	74.7
**ADI** †					
Impoverished*	33473 (15.8)	12.5	8.9	3.7	74.9
No/lower impoverishment	178578 (84.2)	12.6	23.8	8.9	54.7
**RUCC Status** †					
Rural	17261 (8.1)	0.0	5.9	2.9	91.2
Non-metro	66531 (31.4)	0.5	15.8	4.4	79.3
Urban	128259 (60.5)	20.6	26.4	10.6	42.4
**Appalachian** †					
Appalachian	53904 (25.4)	0.3	8.1	10.1	81.4
Not Appalachian	158147 (74.6)	16.8	25.9	7.3	49.9

eHDP: an early-onset hypertensive disorder of pregnancy, where hypertensive symptoms present before 35 weeks; BMI: Body Mass Index; RUCC rural-urban continuum codes, Metal Abbreviations: As: Arsenic, Cd: Cadmium, Cr: Chromium, Hg: Mercury, Pb: Lead, Se: Selenium, Zn: Zinc

*Impoverished Upper quintile of economic deprivation (9–10)

**Imprecise address: Midpoint of street/ City/Zip/ No Geocode,

^†^ Chi-square test statistics <0.05,

Sixteen percent of Hispanic women and 31% of black women had a high probability of concurrent As, Cd, & Pb exposure, and approximately 9% of mothers that experienced eHDP were in this class (Table 3). Twenty-four percent of Hispanic women and 22% of White women had a probability of concurrent exposures to elevated Se & Zn. Additionally, 19% of stillbirth records and 22% of eHDP cases were in this class. The third class, defined by the high probability of elevated Pb & Cr exposure, had the smallest proportion of Black mothers (10%) of any metal exposure class and included 9% of eHDP records. The fourth class, the low exposure group, consisted mostly of women ≤20 years old and almost 60% were White mothers.

**Table 3:** Bivariate and multivariable associations between demographic characteristics, environmental exposures, and class membership.

	eHDP N (%)	PR (95% CI)	aPR (95% CI)	Sensitivity aPR (95% CI)
**Latent class metal exposure**				
Elevated As, Cd & Pb	166 (8.6)	0.64 (0.56, 0.76)	0.70 (0.60, 0.84)	0.62 (0.14, 2.60)
Elevated Se & Zn	418 (21.6)	0.96 (0.86, 1.08)	1.08 (0.96, 1.22)	0.80 (0.52, 1.26)
Elevated Pb & Cr	180 (9.3)	1.12 (0.94, 1.30)	1.22 (1.02, 1.44)	1.48 (0.96, 2.24)
Low Exposure	1168 (60.5)	Reference	Reference	Reference
**Mother’s age (years)**				
≥35	142 (7.3)	1.94 (1.60, 2.34)	1.76 (1.42, 2.18)	2.42 (1.2, 4.92)
29–34	380 (19.7)	1.40 (1.22, 1.60)	1.40 (1.18, 1.66)	1.4 (0.78, 2.52)
24–28	545 (28.2)	1.28 (1.14, 1.46)	1.22 (1.04, 1.40)	1.14 (0.68, 1.88)
21–23	400 (20.7)	1.26 (1.10, 1.46)	1.10 (0.96, 1.28)	1.42 (0.9, 2.24)
≤20	465 (24.1)	Reference	Reference	Reference
**Mother’s race**				
Black	237 (12.3)	1.52 (1.32, 1.74)	1.60 (1.38, 1.86)	2.00 (1.28, 3.16)
Other	76 (3.9)	0.60 (0.48, 0.74)	0.72 (0.56, 0.94)	0.14 (0.02, 1.00)
White	1619 (83.8)	Reference	Reference	Reference
**Mother’s ethnicity**				
Non-Hispanic	1879 (97.3)	1.50 (1.14, 1.96)	1.08 (0.80, 1.48)	2.20 (0.50, 9.60)
Hispanic	53 (2.7)	Reference	Reference	Reference
**Mother’s education**				
College Degree	612 (31.7)	0.92 (0.82, 1.04)	1.10 (0.96, 1.28)	0.62 (0.38, 1.00)
Some College	540 (28.0)	1.14 (1.02, 1.28)	1.20 (1.06, 1.38)	1.04 (0.70, 1.54)
Less than High School	206 (10.7)	0.66 (0.56, 0.78)	0.84 (0.70, 1.02)	0.74 (0.42, 1.30)
Unknown	21.0 (1.1)	1.04 (0.94, 1.14)	1.06 (0.96, 1.20)	1.06 (0.74, 1.56)
High School	553 (28.6)	Reference	Reference	Reference
**Mother’s BMI (kg/m^2^)**				
Obese (≥30.0)	766 (39.6)	2.70 (2.42, 3.02)	2.38 (2.12, 2.66)	2.98 (2.00, 4.44)
Overweight (25.0–30.0)	448 (23.2)	1.56 (1.38, 1.76)	1.48 (1.30, 1.66)	1.94 (1.26, 3.00)
Underweight (<17.9)	46 (2.4)	0.70 (0.52, 0.96)	0.74 (0.54, 1.00)	0.44 (0.10, 1.80)
Unknown	126 (6.5)	3.58 (2.94, 4.34)	3.12 (2.56, 3.82)	6.46 (3.56, 11.66)
Normal (18.0–24.9)	546 (28.3)	Reference	Reference	Reference
**Pre-existing diabetes**				
Yes	87 (4.5)	6.46 (5.18, 8.06)	4.20 (3.34, 5.26)	2.80 (1.08, 7.22)
No or not stated	1845 (95.5)	Reference	Reference	Reference
**Smoking throughout pregnancy**				
No	1654 (85.6)	1.58 (1.16, 2.18)	1.48 (1.06, 2.04)	1.68 (0.56, 5.08)
Unknown	46 (2.4)	1.20 (1.04, 1.38)	1.22 (1.06, 1.42)	1.46 (0.88, 2.46)
Yes	232 (12.0)	Reference	Reference	Reference
Mother married				
Yes	996 (51.6)	1.14 (0.74, 1.78)	1.00 (0.64, 1.58)	0.86 (0.20, 3.76)
No	936 (48.4)	Reference	Reference	Reference
**Stillbirth**				
Yes	56 (2.9)	5.24 (4.00, 6.88)	4.10 (3.10, 5.42)	6.42 (2.66, 15.48)
No or not stated	1876 (97.1)	Reference	Reference	Reference
**Geocoding precision**				
Address point/Street segment	1701 (88.0)	0.88 (0.76, 1.02)	0.96 (0.84, 1.12)	1.12 (0.64, 1.96)
Imprecise**	231 (12.0)	Reference	Reference	Reference
**ADI**				
Impoverished area (9–10)	353 (18.3)	1.20 (1.06, 1.34)	1.04 (0.92, 1.18)	1.46 (0.86, 2.46)
No/lower impoverishment (1–8)	1579 (81.7)	Reference	Reference	Reference
**RUCC Status**				
Rural	169 (8.7)	1.20 (1.02, 1.42)	1.10 (0.9, 1.34)	2.08 (0.64, 6.88)
Non-Metro	720 (37.3)	1.34 (1.22, 1.46)	1.20 (1.08, 1.36)	1.78 (1.20, 2.62)
Urban	1043 (54.0)	Reference	Reference	Reference
**Appalachian**				
Appalachian	599 (31.0)	1.32 (1.20, 1.46)	1.18 (1.04, 1.34)	0.62 (0.36, 1.06)
Not Appalachian	1333 (69.0)	Reference	Reference	Reference

BMI: Body Mass Index, ADI: Area Deprivation Index, RUCC: Rural-Urban Continuum Codes,

**Imprecise: Midpoint of street/ City/Zip/ No Geocode,

Table 3 summarizes the proportion of eHDP cases by covariate and displays the results of the bivariate, multivariable, and sensitivity analyses with posterior probabilities (PPr) of less than 80%. Please see the appendix for counts, percentages, and average PPr of each class for the overall sample and those with a PPr of less than 80%. Of the women who had exposure to trace elements, most women who experienced eHDP had a high probability of elevated exposure to Se & Zn (22%). Women who experienced eHDP were predominantly White (84%), non-Hispanic (97%), and ≤ 20 years old (24%). Approximately 40% were obese, 12% reported smoking throughout their pregnancy, and 52% reported being married.

In the bivariate assessment, women with a high probability of concurrent exposure to As, Cd, & Pb, had a 36% lower prevalence of eHDP (PR = 0.64, 95% CI: 0.56, 0.76) compared to women with a low probability of exposure. In contrast, women with concurrent elevated Pb & Cr exposure had a 12% higher prevalence of eHDP, although this was not a statistically significant difference (PR = 1.12, 95% CI: 0.94, 1.30). Age was also associated with the prevalence of eHDP, with women ≥ 35 years old having twice the prevalence of eHDP compared to those ≤ 20 years old (PR = 1.94, 95% CI: 1.60, 2.34). In addition, mothers with an obese BMI had an eHDP prevalence almost three times higher than those with a normal BMI (PR = 2.70, 95% CI: 2.42, 3.02). Black women also experienced a 52% higher prevalence of eHDP (PR = 1.52, 95% CI: 1.32, 1.74) than White women.

Following covariate adjustment, those in the first latent class with elevated As, Cd, & Pb had a 30% lower rate of eHDP compared to those with low exposure (aPR = 0.70, 95% CI: 0.60 0.84), and those with a high probability of concurrent exposure to elevated Pb & Cr had 22% higher prevalence of eHDP (aPR = 1.22, 95% CI: 1.02, 1.44). The prevalence of eHDP in those ≥35 years old remained high after adjustment, compared to those ≤ 20 years old (aPR = 1.76, 95% CI: 1.42, 2.18). After adjustment, the prevalence of eHDP among those that were obese declined slightly (aPR = 2.38, 95% CI: 2.12, 2.66) but was still two times higher among women with an obese BMI compared to those with a normal BMI. Prevalence estimates were similar among race and smoking throughout pregnancy and among geographic covariates such as geocoding precision and RUCC status. For those with pre-existing diabetes, there was a marked decline in the prevalence of eHDP after adjustment (PR = 6.46, 95% CI: 5.18, 8.06 vs. aPR = 4.20, 95% CI: 3.34, 5.26).

Most estimates in the sensitivity analysis were within the bounds or did not deviate substantially from the adjusted analysis; however, two associations had notable changes. The aPR in the sensitivity analysis had more extreme prevalence ratios for those of unknown BMI than those within a normal BMI range (sensitivity aPR = 2.0, 95% CI 1.28, 3.18), as did the aPR for ADI (aPR = 1.46 95% CI: 0.86, 2.48). The sensitivity analysis for the prevalence of eHDP also had more extreme values than those in the full analysis (sensitivity aPR = 3.12, 95% CI: 2.56, 3.82 compared to aPR = 6.46, 95% CI: 3.56, 11.66).

## Discussion

This study sought to assess geospatial trends of eHDP and visualize the overlap of eHDP clusters in relation to patterns of industrial aerosol emissions of As, Cd, Cr, Hg, and Pb in Kentucky. Using individual-level birth records, we discovered two statistically significant clusters of eHDP, one in Western Kentucky and a second larger cluster in the Appalachian region. Employing an LCA, we identified four subgroups of metal exposures and further detected that women in the latent class with elevated exposure to Pb & Cr had a significantly higher prevalence of eHDP after covariate adjustment. We also found that individual factors such as Black race, maternal age ≥34 years, obesity, and smoking throughout the pregnancy were associated with a higher prevalence of eHDP. Both non-metro women and those who lived within the Appalachian region also had a higher prevalence of eHDP. Our findings contribute to the emerging literature on the association of industrial exposures with HDP, specifically, eHDP.

### Aim 1: Examine the distribution of chemicals of concern across the state

To assess environmental metal exposures, we employed toxicity concentration estimates from RSEI, a promising publicly available population-level dataset that, among other things, estimates the environmental volume of chemicals of concern at small spatial scales (810 m x 810 m grids, CBG, and ZIP codes). Traditionally, to assess the impact of environmental emissions exposure on adverse pregnancy outcomes, studies employ a generalized linear model to individually assess the impact of exposures, adjusting for other important risk factors, on the outcome of interest, with limited (if any) assessment of co-occurrences. Although helpful, these statistical models may not adequately address the impact of interactions, assuming they are additive among various chemicals, which rarely occur in isolation. The RSEI data, which employs consistent methodologies to estimate the local environmental burden of chemicals of concern, also allowed for the exploration of novel methodologies to assess concurrent environmental exposures to chemicals of concern.

Although RSEI data has been infrequently used in health research, preliminary work has found elevated areas with elevated estimates of aerosolized concentrations of Hg and Cd corresponded to higher blood metal levels in children [51]. In a study assessing geospatial patterns and risk factors for preterm birth, Ogneva-Himmelberger and colleagues reported an association between preterm births and RSEI estimated hazard scores—a value that accounts for the size of a given chemical release, the fate of the chemical in the environment, and size and distance of an exposed population [25, 52]. RSEI estimates are based on Toxic Release Inventory (TRI) reports, which are yearly estimates of fugitive and stack emissions self-disclosed by only a subset of facilities in the U.S. Although site-specific characteristics are incorporated if available, generalizations are often made to similar facilities’ characteristics. The RSEI model also does not incorporate non-TRI sources of contamination and does not examine, integrate, or estimate decay products of emissions, which may not have equivalent health risks as the parent product. However, RSEI data offer estimates of environmental concentrations of almost 700 specific contaminants of concern in both air and water (this study focused exclusively on air), adjusted for contaminant characteristics at a spatially confined resolution. Estimates are updated based on the most current data (facilities can correct reported emissions information for up to 3 years) and the most up-to-date methodologies. There is also limited adjustment for meteorological conditions using the EPAs AEROMOD modeling system. Changes in reporting standards have also been relatively few since the program’s inception in 1988, making the data appropriate for longitudinal analyses.

### Aim 2: Identify patterns of exposure to industrial metal emissions and describe the sociodemographic characteristics of mothers in these areas

We employed a latent class model to explore concurrent environmental exposures to identify exposure patterns. The latent class analysis (LCA) creates homogenous and mutually exclusive subgroups using the similarities of response patterns among records [53]. This person-centered approach allows for evaluating complex interactions without sacrificing statistical power, as multiple exposures are combined based on the probability of concurrent exposure.

In this study, we identified four latent classes or patterns of concurrent elevated exposures—As, Cd, & Pb; Pb & Cr; Se & Zn; and none. While most women were not exposed to multiple contaminants of concern (58% of the sample), many women, particularly Black or Hispanic women were. The As, Cd, & Pb exposure class contained 31% of Black women and 16% of Hispanic women. Compared to approximately 8% of White women, 10% of Black women had a high probability of concurrent elevated Pb & Cr exposure. These findings need further scrutiny.

### Aim 3: Evaluate the impact of environmental exposures to industrial metal emissions, adjusting for sociodemographic factors on the risk of eHDP

While there have been limited studies assessing the impact of environmental metal exposures, specifically those identified in our study—Pb and Cr—on eHDP, individual-level assessments suggest a relationship between lead exposure and eHDP [18, 54]. A case-control study of South African women found women with HDP had significantly elevated levels of chromium in pubic hair compared to women in the control group [55]. In our study, we found that although not significant on its own, after adjusting for other covariates, those with a high probability of concurrent exposure to elevated Pb & Cr had a 22% higher prevalence of eHDP compared to those with no/low metal exposures. Further work exploring this relationship is needed.

### Aim 4: Identify areas in the state with a high prevalence of individuals with eHDP

We identified two clusters of eHDP. The first was in Western Kentucky and overlapped with census block groups (CBG) with elevated median estimates of As. The second cluster, located in the eastern, Appalachian region of the state, was larger and had very little visual overlap with the examined metal emissions. These clusters may reflect the geospatial patterns of eHDP risk factors, such as obesity and pre-existing diabetes, particularly elevated in the Appalachian region [49]. However, a future study that assesses samples from these areas, rather than estimates, is needed.

### Strengths and limitations

This study has notable strengths. First, this study employed all primiparous births in Kentucky over ten years (2008–2017). The majority of records geocoded accurately (>90%), and the exposure concentrations were consistently estimated over the entire study period. Howeverthere are significant limitations. Birth certificates may be subject to recall and interviewer bias, particularly with stigmatized health information such as smoking and pre-pregnancy weight.

Exposure classification was also determined using the LCA. This may not accurately reflect patterns in the population, and records may be inappropriately assigned to exposure classifications if they do not have a clear class assignment. Therefore, we conducted a sensitivity analysis for those with low posterior probabilities (<80%) to assess potential bias in latent classes to explore the impact of ambiguous misclassification. Although the point estimates for measures of association were more extreme, most prevalence ratios were in the same direction and had overlapping confidence intervals. However, a few covariates reversed direction (elevated Se & Zn exposure, college education, Appalachian status, and address point/street segment geocoding precision). Overall, this suggests although there was some bias, the impact appears to be limited.

The use of geocoded cross-sectional residential information is also a source of bias. In this study, we determined the CBG based on the geographic coordinates of the geocoded maternal addresses. The CBG was then used to assign exposure status and assess the geospatial clustering of eHDP. Overall, 90% of records geocoded precisely in Kentucky. Inthe non-Appalachian region, the proportion of precise addresses was almost 95%. However, almost 24% of addresses within the Appalachian region geocoded imprecisely to either the midpoint of the street, ZIP code, or city. This geocoding imprecision may also lead to unit-hazard coincidence or the misclassification of CBG characteristics (community characteristics and exposure). and could also result in biased estimates [56]. As the emission patterns of environmental metals were homogenous across the Appalachian region, we do not suspect substantial exposure misclassification. However, we encourage a cautious interpretation of the spatial cluster identified within the Appalachian region. Although we believe that the Appalachian region has a high burden of eHDP, the cluster identified in this study may not be precise. Further study in the areas the clusters were discovered is needed.

Finally, as all information was collected at the time of birth, we could not determine the mother’ ’s length of time at the residential address provided on the birth certificate, nor could we ascertain any residential history [57]. Therefore, if women moved during pregnancy, there may be a misclassification of the exposure and other CBG characteristics [58]. Given the low residential mobility in the Appalachian region, which constitutes 54 of 120 Kentucky’ ’ counties, we expect the misclassification due to moving would be limited [59].

### Future research

In addition to improving the GIS resources within the Appalachian region, future assessments focusing on evaluating a broader range of exposure sources such as drinking water, residential air quality, and occupational exposures would allow for a fuller picture of metal exposures and lead to a better evaluation of health outcomes. Additionally, further exploration of the interaction among toxic exposures would help expand some of the results observed in this study, such as the protective effect of As, Cd, & Pb, but the increased risk for those with a high probability of Pb & Cr exposure.

### Conclusions

This study adds to the limited literature examining the risk of HDP by focusing explicitly on early-onset HDP and employing a latent class methodology to assess multiple environmental exposure patterns. Further, we identified metal exposures, specifically Pb & Cr exposures, as contributing to the prevalence of eHDP. This study suggests that efforts to mitigate metal exposures among women of childbearing age are likely warranted.
